# Utilization of statins and LDL-cholesterol target attainment in Turkish patients with type 2 diabetes - a nationwide cross-sectional study (TEMD dyslipidemia study)

**DOI:** 10.1186/s12944-020-01408-2

**Published:** 2020-11-11

**Authors:** Fahri Bayram, Alper Sonmez, Cem Haymana, Tevfik Sabuncu, Oguzhan Sitki Dizdar, Eren Gurkan, Ayse Kargili Carlioglu, Kemal Agbaht, Didem Ozdemir, Ibrahim Demirci, Cem Barcin, Serpil Salman, Tamer Tetiker, Mustafa Kemal Balci, Nur Kebapci, Canan Ersoy, Volkan Yumuk, Peter P. Toth, Ilhan Satman, TEMD Study Group, Sibel Guldiken, Semra Ayturk, Murat Yilmaz, Mehmet Asik, Nevin Dinccag, Ramazan Cakmak, Fulya Turker, Cemile Idiz, Hulya Hacisahinogullari, Elif Bagdemir, Busra Yildiz, Ozlem Haliloglu, Seda Sancak, Levent Ozsari, Eylem Cagiltay, Oguzhan Deyneli, Eren Imre, Sait Gonen, S. Nur Boysan, Yuksel Altuntas, Feyza Yener Ozturk, Meral Mert, Hamide Piskinpasa, Hasan Aydin, Sazi Imamoglu, Ozen Oz Gul, Sinem Kucuksarac Kiyici, Berrin Cetinarslan, Alev Selek, Teoman Dogru, Ali Kirik, Belgin Efe, Ahmet Kaya, Ilker Cordan, Suleyman Baldane, Cem Onur Kirac, Zehra Capa, Mustafa Cesur, Ilhan Yetkin, Demet Corapcioglu, Sule Canlar, Okan Bulent Yildiz, Suleyman Nahit Sendur, Bekir Cakir, Ahmet Corakci, Mustafa Kutlu, Neslihan Bascil Tutuncu, Yusuf Bozkus, Erman Cakal, Berrin Demirbas, Sibel Ertek, Mustafa Altay, Murat Dagdeviren, Amir Hossein Abedi, Sevki Cetinkalp, Hatice Ozisik, Guzide Gonca Oruk, Serkan Yener, Basak Ozgen Saydam, Engin Guney, Mustafa Unubol, Guzin Fidan Yaylali, Senay Topsakal, Zeliha Hekimsoy, Gulhan Akbaba, Ibrahim Aslan, Sefika Dalkiran, Esen Akbay, Kamile Gul, Muge Ozsan Yilmaz, Emre Bozkirli, Seher Cetinkaya Altuntas, Aysegul Atmaca, Elif Tutku Durmuş, Turkan Mete, Faruk Kutluturk, Ferit Kerim Kucukler, Oguz Dikbas, Safak Akin, Irfan Nuhoglu, Halil Onder Ersoz Halil Onder Ersoz, Taner Bayraktaroglu, Pınar Sisman, Ibrahim Sahin, Sedat Cetin, Ilyas Capoglu, Emin Murat Akbas, Rıfkı Ucler, Mehmet Ali Eren, Alpaslan Kemal Tuzcu, Zafer Pekkolay, Mesut Ozkaya, Mustafa Araz

**Affiliations:** 1 Department of Endocrinology and Metabolism, Erciyes University, School of Medicine, Kayseri, Turkey; 2 Department of Endocrinology and Metabolism, University of Health Sciences, Gulhane School of Medicine, Ankara, Turkey; 3 Department of Endocrinology and Metabolism, University of Health Sciences, Gulhane Training and Research Hospital, Ankara, Turkey; 4 Department of Endocrinology and Metabolism, Harran University, School of Medicine, Sanliurfa, Turkey; 5 Department of Endocrinology and Metabolism, Kayseri Training and Research Hospital, Kayseri, Turkey; 6 Department of Endocrinology and Metabolism, Mustafa Kemal University, School of Medicine, Hatay, Turkey; 7 Department of Endocrinology and Metabolism, Erzurum Training and Research Hospital, Erzurum, Turkey; 8 Department of Endocrinology and Metabolism, Private Defne Hospital, Hatay, Turkey; 9 Department of Endocrinology and Metabolism, Yildirim Beyazit University, School of Medicine, Ankara, Turkey; 10 Department of Cardiology, University of Health Sciences, Gulhane School of Medicine, Ankara, Turkey; 11 Department of Endocrinology and Metabolism, Private Liv Hospital, Istanbul, Turkey; 12 Department of Endocrinology and Metabolism, Cukurova University, School of Medicine, Adana, Turkey; 13 Department of Endocrinology and Metabolism, Akdeniz University, School of Medicine, Antalya, Turkey; 14 Department of Endocrinology and Metabolism, Osmangazi University, School of Medicine, Eskisehir, Turkey; 15 Department of Endocrinology and Metabolism, Uludag University, School of Medicine, Bursa, Turkey; 16 Department of Endocrinology and Metabolism, Istanbul University, Cerrahpasa School of Medicine, Istanbul, Turkey; 17 Ciccarone Center for the Prevention of Cardiovascular Disease, Johns Hopkins University School of Medicine, Baltimore, MD USA; 18 Preventive Cardiology, CGH Medical Center, Sterling, IL USA; 19 Department of Endocrinology and Metabolism, Istanbul University, School of Medicine, Istanbul, Turkey

**Keywords:** Dyslipidemia, Type 2 diabetes mellitus, Lipid-lowering treatments, Physicians’ attitudes, Low-density lipoprotein cholesterol target attainment, Statin cessation, Physician inertia

## Abstract

**Background:**

Attaining acceptable levels of LDL Cholesterol (LDL-C) significantly improves cardiovascular (CV) outcomes in patients with type 2 diabetes mellitus (T2DM). The LDL-C target attainment and the characteristics of patients attaining these targets were investigated in this study. Furthermore, the reasons for not choosing statins and the physicians’ attitudes on the treatment of diabetic dyslipidemia were also examined.

**Methods:**

A nationwide, cross-sectional survey was conducted in tertiary centers for diabetes management. Adult patients with T2DM, who were under follow-up for at least a year in outpatient clinics, were consecutively enrolled for the study. LDL-C goals were defined as below 70 mg/dL for patients with macrovascular complications or diabetic nephropathy, and below 100 mg/dL for other patients. Data about lipid-lowering medications were self-reported.

**Results:**

A total of 4504 patients (female: 58.6%) were enrolled for the study. The mean HbA1c and diabetes duration was 7.73 ± 1.74% and 10.9 ± 7.5 years, respectively. The need for statin treatment was 94.9% (*n* = 4262); however, only 42.4% (*n* = 1807) of these patients were under treatment, and only 24.8% (*n* = 448) of these patients achieved LDL-C targets. The main reason for statin discontinuation was negative media coverage (87.5%), while only a minority of patients (12.5%) mentioned side effects. Physicians initiated lipid-lowering therapy in only 20.3% of patients with high LDL-C levels. It was observed that the female gender was a significant independent predictor of not attaining LDL-C goals (OR: 0.70, 95% CI: 0.59–0.83).

**Conclusions:**

Less than 50 % of patients with T2DM who need statins were under treatment, and only a quarter of them attained their LDL-C targets. There exists a significant gap between the guideline recommendations and the real-world evidence in the treatment of dyslipidemia in T2DM.

**Supplementary Information:**

The online version contains supplementary material available at 10.1186/s12944-020-01408-2.

## Introduction

The risk for atherosclerotic cardiovascular diseases (ASCVD) is very high in patients with type 2 diabetes mellitus (T2DM), occurring 10 years earlier than people without diabetes [1, 2]. Although effective glycemic control prevents microvascular complications [3, 4], it is not sufficient to reduce the cardiovascular outcomes [5, 6]. The regulation of other major cardiovascular risk factors is essential for the reduction of the total cardiovascular risk [7, 8]. Dyslipidemia is one of these major risk factors in patients with T2DM [9, 10]. Low-density lipoprotein cholesterol (LDL-C) levels are not significantly elevated. However, these patients have small and dense LDL particles, increased triglyceride, and reduced high-density lipoprotein cholesterol (HDL-C) levels [11, 12]. These alterations in lipid profiles are characteristic of diabetic dyslipidemia, which is highly atherogenic [11, 12]. The treatment of dyslipidemia is effective both in the primary and secondary prevention of cardiovascular morbidity and mortality [8, 13, 14]. However, many patients with T2DM are not receiving lipid-lowering treatment [15–22], and only a minority of those who are treated attain their lipid targets [16–18, 20, 21, 23].

The data about the lipid target attainment rates in Turkish patients with T2DM is inconsistent [24–26]. Previous studies, which were not representative enough, mentioned LDL-C attainment rates by using higher cut-off levels. **T**urkish Nationwide Surv**E**y of Glycemic and Other **M**etabolic Parameters of Patients with **D**iabetes Mellitus (TEMD Study), has been recently published, showing that LDL-C levels are < 100 mg/dL in only 37% of the patients with T2DM [25]. The present report, TEMD Dyslipidemia Study, further analyzes the TEMD database by defining two LDL targets for patients with high and very high risk. This study is designed to determine the rate of LDL-C target attainment in patients with T2DM according to the hypothesis of statin utilization, and the target LDL-C attainment rates are very low in Turkish patients with T2DM. Sociodemographic and clinical characteristics of patients who attain LDL-C targets, the reasons for not taking or withdrawing from statins, and physicians’ attitudes on the treatment of diabetic dyslipidemia were also assessed in this study.

## Materials and methods

### Study design

The TEMD Dyslipidemia Study has been conducted as a nationwide survey, between 01 April and 30 June 2017. The patients were registered from the tertiary centers throughout Turkey, which were allocated according to the 12 nomenclature of territorial units for statistics regions. The study protocol was approved by the Turkish Ministry of Health, Pharmaceuticals and Medical Devices Agency, Central Ethical Committee (14- MAR- 2017/93189304–514.11.01-E.58933), and registered in ClinicalTrials.gov (NCT 03455101). Informed consent forms were signed by the patients before data collection.

### Study population

**Fig. 1:**
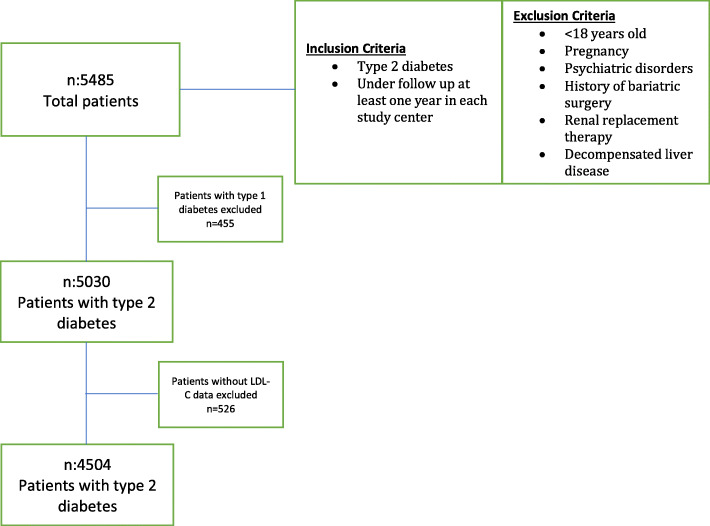
Flow diagram of patient enrolment with inclusion and exclusion criteria

Each study center aimed to consecutively enroll the first 100 patients, who were under follow-up for at least 12 months and meet the inclusion criteria [25]. The present report includes only the data of patients with T2DM (*n* = 4504). The inclusion and exclusion criteria and the flowchart showing patient enrolment is given in Fig. 1.

### Data collection

All participants filled out questionnaires on sociodemographic characteristics, medications, complications, and concomitant diseases. The questionnaires also searched for data on personal diabetes management [diet, exercise, smoking, self-monitoring of blood glucose, and hypoglycemia frequency] and laboratory values. The data on statin usages, such as the history of initiation and discontinuation, the reasons for cessation (side effects, negative effects of the social environment or other), and the percentage of moderate or high potency statins were also evaluated. The introduction of new lipid-lowering medications, as well as changes in their dosing, were also evaluated.

### Anthropometrics and laboratory data

The body mass index (BMI) was calculated by the ratio of weight to the square of height (kg/m^2^). Arterial blood pressure (BP) was recorded in all centers by the automatic BP monitors Omron M2, HEM-7121-E. The measurements were conducted after 5 min rest, and the average of the three consecutive measurements from the same arm was recorded. Patients were also asked to take blood pressure recordings at home twice a day for a week in the sitting position after 5 min of rest. The recordings were noted on the control visits.

All laboratory measurements were conducted in the hospitals where the interviews were performed. The overnight fasting blood samples were taken from the antecubital veins before 10:00 AM. Blood glucose and lipids were measured enzymatically. More specifically, glucose was measured by the hexokinase method in most hospitals, while the glucose oxidase method was used in several centers. Total Cholesterol was measured by cholesterol oxidase, HDL Cholesterol was measured by cholesterol esterase, and Triglycerides were measured by glycerol oxidase methods. Friedewald’s eq. [LDL-C = total cholesterol – (HDL-C + TG/5)] was used for the calculation of LDL-C levels. The formula was used only when the TG was less than 400 mg/dL [27]. Otherwise, LDL-C measurements were performed by the LDL-C measurement kits where available. Glycohemoglobin (HbA1c) measurement was performed by using one of the following methods: High-performance liquid chromatography, turbidimetric inhibition immunoassay, or enzymatic methods.

### Definitions

The definition of dyslipidemia and the treatment targets were taken according to the suggestions of the Clinical Practice Guideline for Diagnosis, Treatment, and Follow-up of Diabetes Mellitus and Its Complications-2017, published by the Society of Endocrinology and Metabolism of Turkey [28], which was prepared according to the ADA-2017 and ESC/EAS-2016 guidelines [29, 30]. Dyslipidemia was defined as TG > 150 mg/dL and/or LDL-C > 100 mg/dL, and/or low HDL-C (men < 40 mg/dL, women < 50 mg/dL), or taking lipid-lowering medications. The target LDL-C level was defined as below 70 mg/dL for very high-risk patients (with macrovascular complications or diabetic nephropathy) and below 100 mg/dL for high-risk patients (without complications). Target TG was defined as < 150 mg/dL, target HDL-C was defined as > 40 mg/dL for men and > 50 mg/dL for women. Patients who attain the target LDL-C levels according to the current guidelines were also analyzed (< 55 mg/dl for very high-risk patients and < 70 mg/dL for high-risk patients) [31]. The following criteria were defined for the patients who need statins: (1) LDL-C levels over 70 mg/dL together with macrovascular complications or diabetic nephropathy; or (2) LDL-C levels over 70 mg/dL in patients over 40 years without any atherosclerotic CVD but with risk factors or markers of target organ damage; or (3) LDL-C levels over 100 mg/dL [29, 30]. A high potency statin was defined as atorvastatin ≥40 mg or rosuvastatin ≥20 mg per day [28–30].

Home BP recordings with values ≥135/85 mmHg or being under antihypertensive treatment was defined as Hypertension. If the patients did not have home BP recordings, the mean office BP ≥ 140/90 mmHg in two different visits was defined as hypertension. Having BMI ≥ 30 kg/m^2^ was defined as Obesity. Treatment targets for glycemia and arterial BP were defined as HbA1c < 7%, home BP < 135/85 mmHg [28, 29]. Performing regular physical activity more than twice per week was regarded as performing regular exercise. Patients having adrenergic symptoms with capillary glucose levels of 70 mg/dL or less were regarded as having hypoglycemia. A high education level was defined as attaining formal education for more than eight years. Macrovascular complications included coronary artery disease, cerebrovascular event, or peripheral artery disease. Additionally, non-palpable extremity pulses, low ankle-brachial index (≤ 0.9), or imaging that revealed established atherosclerotic plaque on coronary or peripheral arteriography, were used to define macrovascular disease. Nephropathy was defined as having albuminuria (≥ 30 mg/g) and/or decreased estimated glomerular filtration rate (eGFR) (< 60 mL/min/1.73 m^2^). Retinopathy was self-reported. Neuropathy was defined by the symptoms of bilateral symmetric distal neuropathy or other autonomous neuropathies attributed to T2DM.

### Statistical analyses

Statistical analyses were performed with SPSS 18.0 (SPSS Inc., Chicago, IL, USA). Data for the categorical variables were expressed as percentages, and continuous variables were expressed as mean ± SD. Continuous variables were compared by using the independent-sample t-test, and the categorical variables were compared by the Chi-square test. The association of LDL-C target attainment with different variables were analyzed by the binominal logistic regression. The inclusion criteria for the model for these variables were the clinical rationale of a potential association with LDL-C target or having a statistical significance (*P* < 0.05) in univariate analyses. The parameters were gender, age (< 65 years vs. older), BMI (< 29.9 kg/m2 vs. ≥30 kg/m2), HbA1c (< 7% vs. ≥7%), BP (< 135/85 mmHg vs. higher), microvascular and macrovascular complications, regular exercise (<=2/week vs. higher), smoking, statin treatment, and being followed up by a private center vs. government hospital. The significance level of a two-tailed *p*-value was < 0.05.

## Results

The sociodemographic and clinical characteristics of patients with T2DM (*n* = 4504, mean age 58.6 ± 10.4 years) are summarized in Table 1. Overall, only 20.5% (*n* = 922) of the total group attained target LDL-C levels. The target attainment rate was 32.7% (*n* = 649) for the high-risk patients (LDL goal < 100 mg/dL), and 10.8% (*n* = 273) for the very high-risk patients (LDL goal < 70 mg/dL). There was a female predominance in patients who did not attain target LDL-C levels (*P* < 0.001). These patients had higher SBP (*P* = 0.03), HbA1c (*P* = 0.015), triglycerides (*P* < 0.001) and HDL-C levels (*P* = 0.005), and had spent less time exercising (*P* < 0.001). The percentage of statin users (*P* < 0.001) and the ratio of being followed clinically in private medical centers (*P* = 0.001) were lower in these patients. These patients had higher micro- and macrovascular complications (*P* < 0.001 for both) and higher obesity rates (*P* = 0.005). Significantly fewer of these patients attained blood pressure targets (*P* = 0.02). When patients were assessed to determine if LDL-C targets recommended in current guidelines [31, 32], only 8.4% (*n* = 152) of patients attained target LDL-C levels.

**Table 1:** Clinical and sociodemographic characteristics of patients with and without on target LDL-C levels

Variables	Total Patients (*n* = 4504)	Patients with LDL-C on target (*n* = 922, 20.5%)	Patients with LDL-C not on target (*n* = 3582, 79.5%)	p
Gender (Female n,%)	2639 (58.6)	475 (51.5)	2164 (60.4)	**< 0.001**
Age (year)	58.6 ± 10.4	58.4 (±10.5)	58.6 (±10.4)	0.68
BMI (kg/m^2^)	32.1 ± 6.5	31.8 (±6.6)	32.1 (±6.4)	0.12
SBP (mmHg)	132.5 ± 18.3	131.4 (±18.1)	132.8 (±18.4)	**0.03**
DBP (mmHg)	80.5 ± 10.7	80.0 (±10.7)	80.6 (±10.7)	0.17
Diabetes duration (year)	10.9 ± 7.5	10.8 (±7.4)	11.0 (±7.5)	0.46
Active smoking (n,%)	561 (12.5)	119 (13.0)	442 (12.4)	0.61
HbA1c (%)	7.73 ± 1.74	7.60 (±1.64)	7.75 (±1.77)	**0.015**
HbA1c levels < 7%	1790 (40.2)	390 (42.7)	1400 (39.5)	0.085
Exercise (n,%)	883 (19.6)	226 (24.7)	657 (18.6)	**< 0.001**
Higher education (n,%)	1722 (38.2)	369 (40.4)	1353 (38.4)	0.26
Private center follow-up (n,%)	483 (10.2)	123 (13.3)	342 (9.5)	**0.001**
Microvascular complications (n,%)	2142 (47.6)	222 (24.1)	1920 (53.6)	**< 0.001**
Retinopathy (n,%)	874 (21.3)	107 (12.5)	767 (23.7)	**< 0.001**
Nephropathy (n,%)	817 (19.6)	84 (9.7)	733 (22.2)	**< 0.001**
Neuropathy (n,%)	1545 (34.7)	163 (17.9)	1382 (39.0)	**< 0.001**
Macrovascular complications (n,%)	1103 (24.5)	165 (17.9)	938 (26.2)	**< 0.001**
Coronary artery disease (n,%)	962 (21.4)	151 (17.7)	811 (24.5)	**< 0.001**
Peripheral artery disease (n,%)	175 (4.2)	28 (3.2)	147 (4.4)	0.11
Cerebrovascular disease (n,%)	132 (3.0)	19 (2.1)	113 (3.3)	0.07
Obesity (n,%)	2593 (58.3)	472 (51.7)	2027 (56.8)	**0.005**
Hypertension (n,%)	3074 (68.6)	621 (67.8)	2453 (68.8)	0.58
Blood pressure on target (n,%)	3128 (69.9)	668 (73.1)	2460 (69.1)	**0.020**
LDL-Cholesterol (mg/dl)	113.9 (±36.2)	73.8 (±17.1)	124.2 (±32.4)	**< 0.001**
HDL-Cholesterol (mg/dl)	46.5 (±12.9)	45.5 (±14.7)	46.9 (±12.4)	**0.005**
HDL- Cholesterol on target (n,%)	1978 (43.9)	376 (41.8)	1602 (46.8)	**0.007**
Triglycerides (mg/dl)	182.1 (±128.7)	164.0 (±113.7)	181.4 (±115.8)	**< 0.001**
Triglycerides on target (n,%)	2272 (50.4)	529 (57.8)	1743 (48.8)	**< 0.001**
Statin treatment (n,%)	1807 (40.1)	448 (48.6)	1359 (37.9)	**< 0.001**
High intensity statin (n,%)	181 (4.0)	41 (22.7)	140 (77.3)	0.48

*BMI* body mass index, *SBP* systolic blood pressure, *DBP* diastolic blood pressure, *HbA1c* glycosylated hemoglobin, *LDL* low-density lipoprotein, *HDL* high-density lipoprotein, Data are expressed as (mean ± SD) number (%) where appropriate

*p*-values are derived from Student’s t-test and Chi-square tests for continuous and categorical variables, respectively

**Fig. 2:**
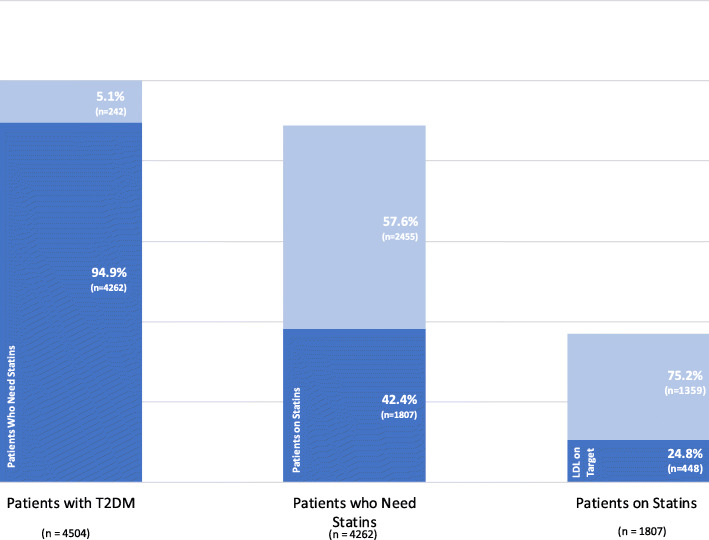
Distribution of patients in terms of statin use. T2DM, type 2 diabetes mellitus; LDL, low-density lipoprotein

The percentage of patients who qualified for statin treatment was 94.9% (*n* = 4262). However, only 44.8% (*n* = 1807) of these patients received statin treatment. The statin utilization rate was 32.2% (*n* = 640) for the high risk and 46.3% (*n* = 1167) for the very-high risk population. Only 24.8% (*n* = 448) of the patients who were on treatment, achieved the target LDL-C levels (Fig. 2).

**Fig. 3:**
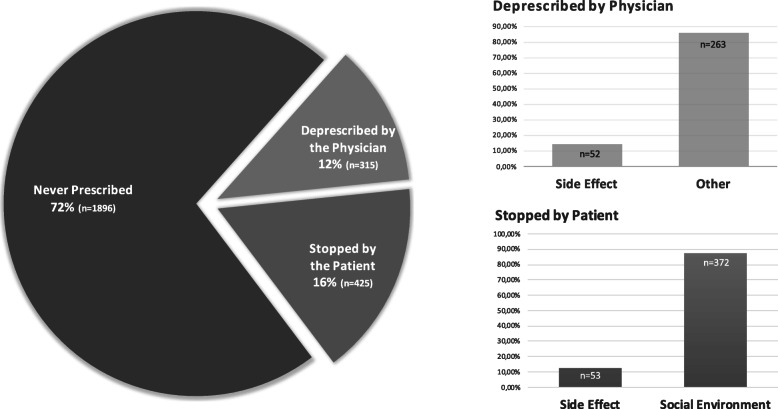
The reasons of not taking lipid lowering treatment in patients with T2DM

Only 10% of the patients on statin treatment were taking high potency statins. Most of the patients who were not taking statins had never been advised to do so (71.9%, *n* = 1869). Others were formerly treated, but treatment was subsequently withdrawn (28.1%, *n* = 740). Of these patients, 42.6% (*n* = 315) had their statins discontinued by their physician, while 57.4% (*n* = 425) chose to discontinue statin therapy of their own accord. Patients who discontinued statins commonly addressed the negative effects of social environment and media coverage (*n* = 372, 87.5%), while only a minority (*n* = 53, 12.5%) invoked the side effects of statins as being the cause of discontinuing these medications (Fig. 3). Most of the patients (*n* = 263, 85.7%) who discontinued statins according to the recommendations of the healthcare providers did not experience significant adverse side effects (Fig. 2). Physicians prescribed new lipid-lowering medications or changed the dosages of ongoing medications in only 20.3% (*n* = 727) of patients unable to achieve target LDL-C levels.

**Fig. 4:**
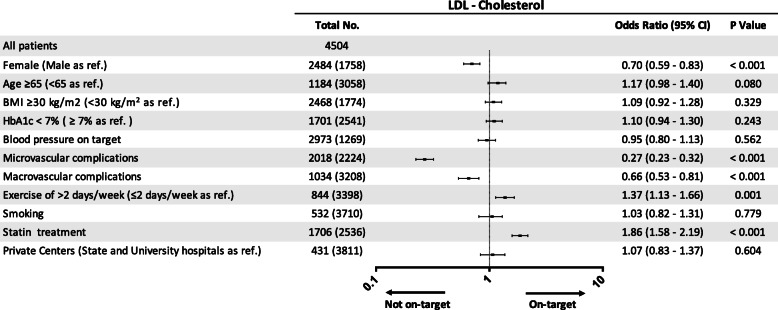
Multivariate analysis of factors associated with LDL-C goal attainment. BMI, body mass index; HbA1c, glycosylated hemoglobin

According to the multivariate analyses, the variables independently associated with risk-stratified LDL-C target attainment were the female gender (OR: 0.70, 95% CI: 0.59–0.83), microvascular (OR: 0.27, 95% CI: 0.23–0.32), and macrovascular complications (OR: 0.66, 95% CI: 0.53–0.81), performing exercise (OR: 1.37, 95% CI: 1.13–1.66), and taking statins (OR: 1.86, 95% CI: 1.58–2.19) (Fig. 4).

## Discussion

The results of the TEMD Dyslipidemia study show that almost 80% of patients with T2DM do not attain LDL-C target levels in Turkey. Less than half of patients who need statins are under treatment and only a quarter of them attain LDL-C targets. Most of the patients who are not taking statins report that they were not advised to take statins by their physicians previously. The main reason for statin cessation is not the risk for adverse side effects, but rather the negative thoughts spread socially or by the media. The TEMD Dyslipidemia study also shows that female sex, receiving statin treatment, performing exercise, and having micro and macrovascular complications are independent predictors of LDL-C target attainment. Finally, it appears that Turkish physicians are not eager to prescribe statins to patients with diabetes mellitus.

The CVD risk is significantly increased in patients with T2DM [1, 2]. In order to establish risk reduction, not only glycemia levels, but also other cardiovascular risk factors should be controlled [9]. Dyslipidemia is prevalent in patients with T2DM [10], and attaining target lipid levels significantly reduces the risk of major cardiovascular outcomes in these patients [8, 13, 14]. However, reports from different regions of the world show insufficient LDL-C attainment rates [16, 17, 20, 21, 23]. One of the main reasons for this low level of target attainment may be the different risk states of the patients involved in these reports. It appears that people with diabetes having CVD or chronic kidney disease (CKD), achieve LDL-C targets much better than those without any comorbidities [17, 20, 23]. Among the different risk groups, people with recent acute coronary syndromes have the highest LDL-C goal attainment rates [17, 20]. The differences between the goal attainment rates are also related to the various definitions of LDL-C targets. The highest LDL-C target attainment rates in patients with diabetes are reported in Holland (%56) [16, 33]. However, the LDL-C targets in these reports were taken as ≤100 mg/dL both for the high-risk and very high-risk patients, in accordance with the Dutch lipid guidelines [34]. Finally, the progressive lowering of the LDL-C targets in recent years may be another reason for the discordance between the reports. Previous reports from Turkey mention the LDL-C target attainment rates between 15 to 25% [26, 35–37]. However, the target LDL-C was defined as < 100 mg/dl in all of these studies. In the current study, a lower LDL-C target was defined for very high-risk patients. This is probably the reason for relatively low achievement rates of the LDL-C targets (20.5% in total; 32.7% high risk and 10.8% very-high risk groups) in the TEMD Dyslipidemia study. This report is prepared before the publication of the recent dyslipidemia guidelines, which increasingly recommend much lower LDL-C targets in patients with T2DM [31, 38]. When study patients were reanalyzed according to current LDL-C goals, less than 10% of them were found to attain the LDL-C targets (9.6% high risk and 4.7% very-high). Overall, these data point to a significant disparity between the recommendations of the guidelines and real-world evidence in the management of diabetic dyslipidemia.

Statins are the most effective treatment for dyslipidemia, both in primary and secondary CVD prevention, as well as in patients with diabetes [8, 13]. Reports from different regions of the world show varying statin utilization rates, ranging from 20 to 70% [15–22]. One of the main reasons for the variations may be the different risk categories of the patients. Patients without CVD have significantly lower statin utilization rates, while the rates are between 41 and 70% in those with CVD [17, 19, 20, 22, 33]. However, the statin utilization rates of patients with similar cardiovascular risk states are also different in various regions of the world. Recent literature from the Netherlands and UK mention that about 66–70% of patients with diabetes without CVD are receiving statin treatment [16, 19], while the numbers from Germany, USA, China, and Japan are lower, ranging between 22 to 42% [17, 18, 21, 22] . The reason for the disparities of these countries with comparable socioeconomic status is not clear. Finally, increasing awareness about the advantages of statins may improve the statin utilization rates in time. According to reports from the USA, the statin utilization rates in patients with similar risk categories almost doubled in ten years, from 21 to 40% [22, 39]. According to the results of the present study, the 45% statin prescription rate (32.2% for the high risk and 46.3% for the very-high risk groups) in Turkey is similar to the rates reported in studies from countries such as Japan, China, Germany, and the USA [17, 20–22]. The results of the current study show that the statin prescription rates are increasing in Turkey. Previous reports mentioned only 20 to 33% of patients with diabetes were receiving statin treatment in Turkey [35, 37]. However, regarding the current recommendations on aggressive lipid therapy, the current rates of statin therapy in patients with diabetes are far from adequate. The current study shows that statins have never been prescribed previously in two-thirds of patients who are not currently being treated with a statin. It should be emphasized that the patients reported upon herein have been followed for at least one year in tertiary diabetes care units. Even in this selected patient population, it appears that physicians largely ignore statins and other lipid-lowering medications.

The TEMD Dyslipidemia study shows that statins were withdrawn in one-third of patients. The most important reason for statin cessation is not the side effects, but rather the negative effects of the media social environment. Negative campaigns on statins in media are effective throughout the world. It has also been shown in other populations that patients are likely to stop taking statins after negative media coverage [40, 41]. Negative campaigns against statins is a prominent issue for statin cessation in Turkey. In a previous survey, high rates of statin cessation was reported in patients with familial hypercholesterolemia due to the negative environmental effects [42]. The TEMD Dyslipidemia Study is the first nationwide study in Turkey, showing the link between negative news stories and the statin cessation rate in patients with T2DM. Negative media coverage appears to be a significant obstacle to the successful management of dyslipidemia, as statin discontinuation is associated with a significant increase in the risk of cardiovascular mortality and morbidity [43]. This problem may also influence doctors’ decisions on statins and their trust in the medical literature as well [44]. Regarding the medical records of the TEMD Study, physicians prescribe or increase the dosages of lipid-lowering drugs only in one-fifth of patients with high LDL-C levels. Unfortunately, the present report does not give us details about the reasons for the physicians’ inertia for the management of dyslipidemia in patients with T2DM. Previous reports point out the desire to avoid potential side effects or the lack of awareness of the guideline recommendations as the reasons for the physicians’ inertia in dyslipidemia treatment [45].

The TEMD Dyslipidemia study allowed us to better define patients who have uncontrolled LDL-C levels. These patients were predominantly women, they did not exercise, and they were likely to have obesity and macro- or microvascular complications. The effect of obesity on the pathogenesis of dyslipidemia is clearly defined [46], and the role of regular physical activity on achieving the LDL-C target is well reported [47]. However, why female sex is disadvantageous for the attainment of LDL-C targets is not clearly known. Reports from different countries also show that men are more likely to have adequate lipid control than women [18, 48, 49]. Possible explanations of the gender disparity may be the lower adherence to medications and lower prescription of statins among women [48–51]. Also, side effects of statins, especially muscle symptoms, occur more frequently in women, which may increase the statin cessation risk [52]. Women in Turkey often have a home dependent life, perform lesser physical activity than men, and have a higher prevalence of obesity [53]. These are the other possible factors decreasing the LDL-C target achievement rates in women. The TEMD Study also showed that micro- or macrovascular complications are negatively associated with the achievement of LDL-C targets. In the present study, it was found that patients with macro and microvascular complications have higher statin utilization rates than patients without complications (57% vs. 35%, respectively). The reason for the lower rates of achieving LDL-C targets despite the higher utilization of statins in complicated patients is probably to target lower LDL-C levels in these patients [28, 31, 38]. The negative thoughts of patients on statins and the physicians’ inertia might mutually prevent the use of higher dosages of statins in these patients with micro- or macrovascular complications.

## Study strength and limitations

The enrollment of large numbers of patients and multicenter design are the notable strengths of the TEMD Dyslipidemia study. However, there are several limitations. First, the cross-sectional design of the study may preclude causal relationships between predictive factors and the achievement of LDL-C targets. Moreover, as all the enrolled patients were followed-up in tertiary endocrine or diabetes units, they were more likely to have multiple comorbidities and complications. The data about the lack of or withdrawal of lipid-lowering medications and presence of retinopathy were obtained by the interviews with patients, which may not be reliable. Also, the adherence of patients to lipid-lowering drugs was not evaluated. Finally, the physicians’ attitudes on lipid-lowering medications are not investigated in detail.

## Conclusion

In conclusion, the TEMD Dyslipidemia study points out a significant gap between the recommendations of the guidelines and the real-world experience in Turkey. Only one in every five patients with diabetes achieves risk-stratified LDL-C targets even in the tertiary care centers for diabetes management. The negative media coverage appears to be the most common cause of statin withdrawal, and the physicians’ inertia to prescribe lipid-lowering drugs is prominent. Overall, these data point out that urgent measures should be taken to improve diabetes care in Turkey. In order to establish a nationwide policy change; it is extremely important to lead physicians by emphasizing the importance of lipid-lowering therapy on the cardiovascular outcomes of patients with diabetes. Also, public opinion should be affected by constantly educating patients about the evidence-based data and positive effects of statins in the lay media. Finally, further prospective studies should be implemented to observe the effects of these policy changes on patient outcomes.

## Supplementary Information

**Additional file 1.**

## Data Availability

Data are available from the authors on request.

## Abbreviations

ASCVD: Atherosclerotic cardiovascular diseases
BP: Blood pressure
BMI: Body mass index
CKD: Chronic kidney disease
CVD: Cardiovascular disease
eGFR: Estimated glomerular filtration rate
HbA1c: Glycohemoglobin
HDL-C: High-density lipoprotein cholesterol
LDL-C: Low-density lipoprotein cholesterol
TEMD Study: **T**urkish Nationwide Surv**E**y of Glycemic and Other **M**etabolic Parameters of Patients with **D**iabetes Mellitus
T2DM: Type 2 diabetes mellitus.
