# Supplementary material for: Utilization of statins and LDL-cholesterol target attainment in Turkish patients with type 2 diabetes - a nationwide cross-sectional study (TEMD dyslipidemia study)
Source: Lipids Health Dis. 2020 Nov 11;19:237. doi: 10.1186/s12944-020-01408-2 (PMC7659134; doi:10.1186/s12944-020-01408-2)
Supplement: Supplementary file 1 — Additional file 1. [file 12944_2020_1408_MOESM1_ESM.docx]

**SUPPLEMENTARY DATA**

| TEMD survey study centers | | Names of the researchers |
| --- | --- | --- |
| Trakya University, School of Medicine, Department of Endocrinology and Metabolism | | Sibel GULDIKEN, Semra AYTURK |
| Corlu REYAP Private Hospital, Department of Endocrinology and Metabolism | | Murat YILMAZ |
| Canakkale 18 March University, School of Medicine, Department of Endocrinology and Metabolism, | | Mehmet ASIK |
| Istanbul University, School of Medicine, Department of Endocrinology and Metabolism, | | Nevin DINCCAG, Ramazan CAKMAK, Fulya TURKER, Cemile IDIZ, Hulya HACISAHINOGULLARI, Elif BAGDEMİR, Busra YILDIZ |
| Istanbul University, Cerrahpasa , School of Medicine, Department of Endocrinology and Metabolism, | | Ozlem HALILOGLU |
| University of Health Sciences, School of Medicine, Fatih Sultan Mehmet Training and Research Hospital, Department of Endocrinology and Metabolism | | Seda SANCAK |
| University of Health Sciences, School of Medicine, Sultanabdulhamit Training and Research Hospital, Department of Endocrinology and Metabolism | | Levent OZSARI, Eylem CAGILTAY |
| Marmara University, School of Medicine, Department of Endocrinology and Metabolism | | Oguzhan DEYNELI, Eren IMRE |
| İstanbul Science University, School of Medicine, Department of Endocrinology and Metabolism | | SAIT GONEN, S.Nur BOYSAN |
| University of Health Sciences, School of Medicine, Sisli Hamidiye Etfal Training and Research Hospital, Department of Endocrinology and Metabolism | | Yuksel ALTUNTAS, Feyza Yener OZTURK |
| University of Health Sciences, School of Medicine, İstanbul Bakırkoy Dr. Sadi Konuk Training and Research Hospital, Department of Endocrinology and Metabolism | | Meral MERT, Hamide PISKINPASA |
| Yeditepe University, School of Medicine, Department of Endocrinology and Metabolism | | Hasan AYDIN |
| Private Office | | Sazi IMAMOGLU |
| Uludag University, School of Medicine, Department of Endocrinology and Metabolism | | Ozen OZ GUL |
| University of Health Sciences, School of Medicine, Bursa Sevket Yılmaz Training and Research Hospital, Department of Endocrinology and Metabolism | | Sinem KUCUKSARAC  KIYICI |
| Kocaeli University, School of Medicine, Department of Endocrinology and Metabolism | | Berrin CETINARSLAN, Alev SELEK |
| Balıkesir University, School of Medicine, Department of Internal Medicine | | Teoman DOGRU, Ali KIRIK |
| Eskisehir Osmangazi University, School of Medicine, Department of Endocrinology and Metabolism | | Belgin EFE |
| Necmettin Erbakan University, School of  Medicine, Department of Endocrinology and  Metabolism | Ahmet KAYA, Ilker CORDAN | |
| Selcuk University, School of Medicine,  Department of Endocrinology and Metabolism | Suleyman BALDANE, Cem Onur KIRAC | |
| University of Health Sciences, Gulhane School of Medicine and Gulhane Training and Research Hospital, Department of Endocrinology and Metabolism | Zehra CAPA | |
| Private GUven Hospital, Department of  Endocrinology and Metabolism | Mustafa CESUR | |
| Gazi University, School of Medicine, Department  of Endocrinology and Metabolism | Ilhan YETKIN | |
| Ankara University, School of Medicine,  Department of Endocrinology and Metabolism | Demet CORAPCIOGLU, Sule CANLAR | |
| Hacettepe University, School of Medicine,  Department of Endocrinology and Metabolism | Okan Bulent YILDIZ, Suleyman Nahit  SENDUR | |
| Yıldırım Beyazıt University, School of  Medicine, Department of Endocrinology and  Metabolism | Bekir CAKIR | |
| Ufuk University, School of Medicine, Department of Endocrinology and Metabolism | Ahmet CORAKCI | |
| Private Bayındır Hospital, Department of Endocrinology and Metabolism | Mustafa KUTLU | |
| Baskent University, School of Medicine, Department of Endocrinology and Metabolism | Neslihan BASCIL TUTUNCU, Yusuf  BOZKUS | |
| University of Health Sciences, School of Medicine, Dıskapı Yıldırım Beyazıt Training and Research Hospital, Department of Endocrinology and Metabolism | Erman CAKAL | |
| TOBB University, School of Medicine, Department of Endocrinology and Metabolism | Berrin DEMIRBAS | |
| Private Memorial Hospital, Department of Endocrinology and Metabolism | Sibel ERTEK | |
| University of Health Sciences, School of Medicine, Kecioren Training and Research Hospital, Department of Endocrinology and Metabolism | Mustafa ALTAY, Murat DAGDEVIREN | |
| Erciyes University, School of Medicine, Department of Endocrinology and Metabolism | Amir Hossein ABEDI | |
| Ege University, School of Medicine, Department of Endocrinology and Metabolism | Sevki CETINKALP, Hatice OZISIK | |
| University of Health Sciences, School of Medicine, İzmir AtatUrk Training and Research Hospital, Department of Endocrinology and Metabolism | Guzide Gonca ORUK | |
| Dokuz Eylul University, School of Medicine, Department of Endocrinology and Metabolism | Serkan YENER, Basak Ozgen SAYDAM | |
| Adnan Menderes University, School of Medicine, Department of Endocrinology and Metabolism | Engin GUNEY, Mustafa UNUBOL | |
| Pamukkale University, School of Medicine, Department of Endocrinology and Metabolism | Guzin Fidan YAYLALI, Senay TOPSAKAL | |
| Celal Bayar University, School of Medicine, Department of Endocrinology and Metabolism | Zeliha HEKIMSOY | |
| Mugla University, School of Medicine, Department of Endocrinology and Metabolism | Gulhan AKBABA | |
| University of Health Sciences, School of Medicine, Antalya Training and Research Hospital, Department of Endocrinology and Metabolism | Ibrahim ASLAN | |
| Akdeniz University, School of Medicine, Department of Endocrinology and Metabolism | Sefika DALKIRAN | |
| Mersin University, School of Medicine, Department of Endocrinology and Metabolism | Esen AKBAY | |
| Kahramanmaras Sutcu İmam University, School of Medicine, Department of Endocrinology and Metabolism | Kamile GUL | |
| Mustafa Kemal University, School of Medicine, Department of Endocrinology and Metabolism | Muge Ozsan YILMAZ | |
| Baskent University, Adana Training Hospital, Department of Endocrinology and Metabolism | Emre BOZKIRLI | |
| Cukurova University, School of Medicine, Department of Endocrinology and Metabolism | Seher CETİNKAYA  ALTUNTAS | |
| 19 Mayıs University, School of Medicine, Department of Endocrinology and Metabolism | Aysegul ATMACA, Elif Tutku DURMUS | |
| University of Health Sciences, School of Medicine, Samsun Training and Research Hospital, Department of Endocrinology and Metabolism | Turkan METE | |
| Gaziosmanpasa University, School of Medicine, Department of Endocrinology and Metabolism, | Faruk KUTLUTURK | |
| Hitit University, School of Medicine, Department of Endocrinology and Metabolism | Ferit Kerim KUCUKLER | |
| Giresun University, School of Medicine, Department of Endocrinology and Metabolism | Oguz DIKBAS | |
| Recep Tayyip Erdogan University, School of Medicine, Department of Endocrinology and Metabolism | Safak AKIN | |
| Karadeniz Technical University, School of Medicine, Department of Endocrinology and Metabolism | Irfan NUHOGLU, Halil Onder ERSOZ | |
| Bulent Ecevit University, School of Medicine, Department of Endocrinology and Metabolism | Taner BAYRAKTAROGLU | |
| Kars Harakani State Hospital, Department of Endocrinology and Metabolism | Pınar SISMAN | |
| İnonu University, School of Medicine, Department of Endocrinology and Metabolism | Ibrahim SAHIN, Sedat CETIN | |
| Erzincan University, School of Medicine, Department of Endocrinology and Metabolism | Ilyas CAPOGLU, Emin Murat AKBAS | |
| Yuzuncu Yıl University, School of Medicine, Department of Endocrinology and Metabolism | Rıfkı UCLER | |
| Harran University, School of Medicine, Department of Endocrinology and Metabolism | Mehmet Ali EREN | |
| Dicle University, School of Medicine, Department of Endocrinology and Metabolism | Alpaslan Kemal TUZCU, Zafer PEKKOLAY | |
| University of Health Sciences, School of Medicine, Gaziantep Ersin Arslan Research and Training Hospital | Mesut OZKAYA | |
| Gaziantep University, School of Medicine, Department of Endocrinology and Metabolism | Mustafa ARAZ | |
